# Pinning down social vulnerability in Sindh Province, Pakistan: from narratives to numbers, and back again

**DOI:** 10.1111/disa.12315

**Published:** 2018-11-19

**Authors:** Daanish Mustafa, Giovanna Gioli, Manzoor Memon, Meher Noshirwani, Iffat Idris, Nadeem Ahmed

**Affiliations:** ^1^ Reader in the Department of Geography, King's College London, United Kingdom; ^2^ Lecturer at the Institute of Geography, University of Edinburgh, United Kingdom; ^3^ Independent consultant, Pakistan; ^4^ Independent scholar, Pakistan; ^5^ Independent scholar, United Kingdom; ^6^ Social Policy Advisor in the Federal SDG (Sustainable Development Goals) Support Unit, Ministry of Planning Development and Reform, Government of Pakistan.

**Keywords:** gender, Pakistan, political economic factors, Sindh, vulnerabilities and capacities index, vulnerability

## Abstract

This paper reflects critically on the results of a vulnerability assessment process at the household and community scale using a quantitative vulnerabilities and capacities index. It validates a methodology for a social vulnerability assessment at the local scale in 62 villages across four agro‐ecological/livelihood zones in Sindh Province, Pakistan. The study finds that the move from vulnerability narratives to numbers improves the comparability and communicational strength of the concept. The depth and nuance of vulnerability, however, can be realised only by a return to narrative. Caution is needed, therefore: the index can be used in conjunction with qualitative assessments, but not instead of them. More substantively, the results show that vulnerability is more a function of historico‐political economic factors and cultural ethos than any biophysical changes wrought by climate. The emerging gendered vulnerability picture revealed extremes of poverty and a lack of capacity to cope with contemporary environmental and social stresses.

## Introduction

**Figure 1:**
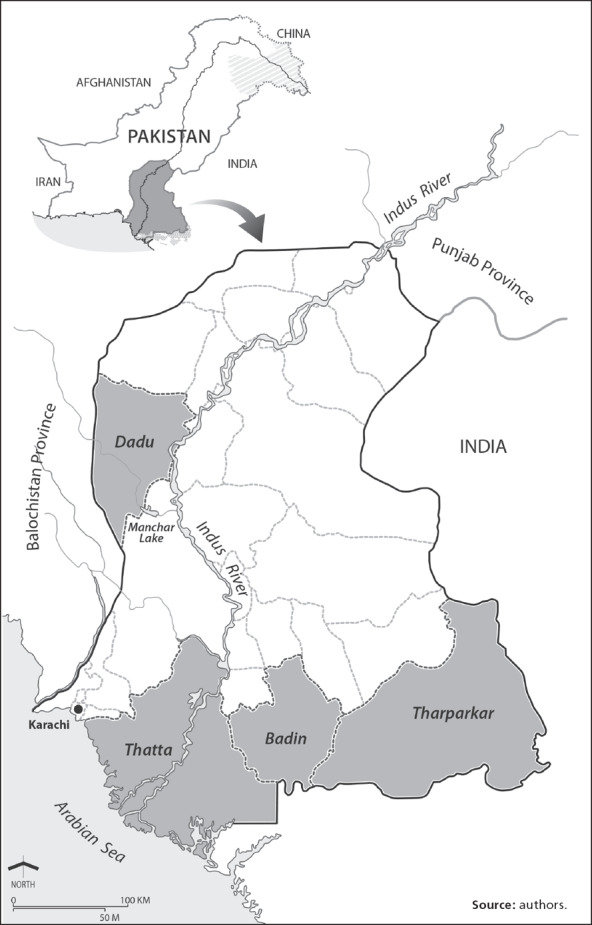
A general map of Sindh Province and the four study districts

Vulnerability is a foundational concept within the hazards tradition and its more current offshoot fields of disaster risk reduction (DRR) and climate change adaptation (Bassett and Fogelman, 2013). The concept has multiple definitions and conceptualisations, within which it is broadly understood either as an outcome of a biophysical threat (see, for example, Burton, Kates, and White, 1978) or as a result of politico‐economic factors and one's social position (O'Keefe et al., 1976). This paper engages with the concept in its politico‐economic sense. When it comes to climate vulnerability, though, the question of future biophysical climate stress often occludes the present‐day politico‐economic factors driving vulnerability (see, for example, Tyler and Moench, 2012). Ambivalence about the drivers and the importance of social vulnerability to climate change may also be a function of uncertainty about how to measure it and to incorporate it better in policy. This paper validates a methodology for measuring social vulnerability and reflects critically on the results of a vulnerability assessment study undertaken in 62 villages across four agro‐ecological/livelihood zones in the four districts of Sindh Province, Pakistan (see Figure 1).

Vulnerability is understood here to mean susceptibility to damage caused by environmental extremes owing to one's social position, as well as the relative inability to recover from that damage (Cutter, 1996; Mustafa, 1998; Adger, 2006). It is embedded in everyday power relations and the political economy, inflected by class (Pelling, 1998; Mustafa, 2005), ethnicity (Bolin, 2007), and gender (Sultana, 2010), among other things. Hence, vulnerability is highly contingent on the social context, and is dynamic and coproduced through evolving power and social relations.

The research community has not been very successful in imparting the full import of the concept to the policy realm, in part because of policymakers' lack of affinity with nuanced qualitative information. Consequently, the study ventured to use a vulnerabilities and capacities index (VCI), following Mustafa et al. (2011), to assess the variance in household‐ and community‐level vulnerability, and to see if the numbers could be validated by the more customary—at least in the critical realist and political‐ecological tradition—qualitative information (see, for example, Mustafa, 2002; Robbins, 2004). The move from vulnerability narratives to numbers is motivated primarily by recognition that quantitative information is likely to be more comparable across contexts than is qualitative information, and, furthermore, will provide a simpler tool for decision‐making.

The quantitative and qualitative vulnerability assessment results presented here paint a picture of differential household‐ and community‐level vulnerability. The policy implications of being able to plot social vulnerability quantitatively and visually are obvious, including for resource allocation, targeted interventions, and even disaster relief. The quantitative vulnerability index calculations, along with the qualitative information, also draw attention to the attitudinal institutional and material drivers of vulnerability. Those drivers, as will be demonstrated by the vulnerability assessment results, are in fact embedded in the political economic context of Pakistan, and are very loosely related to the biophysical risks that emerge from climate change. The vulnerability assessment, following Wescoat, Jr. (1991) and Taylor (2015), thus points up the importance of focusing on the social drivers of vulnerability in the present, as the best strategy to adapt to future climate change, as well as contemporary environmental hazards.

## Geographical research context

Pakistan, like most countries in the Global South, is experiencing a socioeconomic transition, principally from purely rural and urban livelihoods to mixed urban/rural (*desakota*—literally meaning country and town) livelihoods (The Desakota Study Team, 2008). Existing hydro‐climatic regimes coupled with human social systems present formidable enough challenges to securing livelihoods, ecosystem services, and welfare gains (Mustafa, 2013). The challenges will probably become even more formidable via double exposure to climatic and system socioeconomic change (Leichenko and O'Brien, 2008). The four districts of Dadu, Thatta, Badin, and Tharparkar were chosen with the above context in mind. Table 1 illustrates the distribution of villages by agro‐ecological/livelihood zone.

**Table 1:** Number of villages included in the study by agro‐ecological/livelihood zone

Agro‐ecological zone	Badin	Dadu	Tharparkar	Thatta	Total
Canal irrigated (fresh groundwater)	2	5	0	4	**11**
Canal irrigated (saline groundwater)	12	7	2	8	**29**
Agro‐pastoral	0	3	13	0	**16**
Fishing	1	2	0	3	**6**
**Total**	**15**	**17**	**15**	**15**	**62**

**Source:** authors.

Sindh is the lower part of the Indus River system's surface irrigation system. Most of the population density as well as the agricultural productivity of the province is based on canal irrigation. Canal irrigated villages are, however, divided into two zones: those with fresh groundwater; and those with saline groundwater. The villages with fresh groundwater have the choice of supplementing inherently scarce canal water with groundwater irrigation, whereas those with saline groundwater do not. This is important in terms of the choice of crops and the sustainability of livelihoods in the two zones. The non‐canal irrigated regions of Sindh invariably centre on pastoral or agro‐pastoral livelihood systems, where mostly itinerant communities spend the dry season grazing animals, such as camels and goats, and cultivate a crop during the rainy season using the leftover moisture, from whatever precipitation has occurred. This classification captures the issues facing villages in those categories. Finally, the category of riverine and estuarine fishing communities captures the vulnerability profile of these much neglected groups within this eco‐livelihood system.

Coastal and riverine flooding, droughts, and heat stress are some of the common hazards in the study villages. Formal warning systems exist only for coastal and riverine flooding. Even in this case, though, the warning system entails facsimile messages from the Pakistan Meteorological Department (PMD) to government functionaries who may pass on the information orally to their subordinates, who in turn may or may not convey it to the general populace using local police stations or mosques (Mustafa and Wescoat, Jr., 1997; Mustafa et al., 2015). Vernacular warning systems, meanwhile, depend on indigenous knowledge and sometimes are more equitably accessible from a gender standpoint (Mustafa et al., 2015).

Approximately 20 households in each village were randomly selected for inclusion in the survey. Two adult members from each household, one man and one woman, were interviewed to ensure sex‐disaggregated data. Despite the patriarchal norms of rural Sindhi society, the household data were further disaggregated into men‐headed households (MHHs) (n=1,102) and women‐headed households (WHHs) (n=157). This classification, while problematic, as it may legitimise typically male‐dominated, hierarchical power structures at the household level (Buvinic and Gupta, 1997), was nevertheless useful in understanding gendered vulnerability. In addition to the questionnaires, two focus group discussions (FGDs), one with males and one with females, were also held in each of the study villages. The FGDs were conducted by local facilitators with the research team from the Social Policy and Development Centre (SPDC) in attendance and taking notes during the proceedings. The findings of the two sources were rechecked via shared learning dialogues (Moench and Dixit, 2007) with key informants, representatives of government and civil society, and decision‐makers in each of the districts to get their perspectives. The development of the questionnaire was influenced above all by concern to test the capability of the VCI to capture variance in vulnerability across and within the analytical categories.

## The vulnerabilities and capacities index

Most social vulnerability research of the past has been based on qualitative information presented as narratives to capture the complexities, linkages, and nuances contributing to differential patterns of damage (see, for example, Cutter, Mitchell, and Scott, 2000; Halvorson, 2003; Collins, 2009; Murray, 2009). In the policy world, however, it is very rare for textual material to be the basis for action. Most decision‐makers are looking for concise, preferably quantitative information that is generalisable to larger populations and can help with ranking and prioritising target populations and activities, respectively. As Hinkel (2011) argues, though, vulnerability assessments are more appropriately performed at local scales where systems can be narrowly defined and contain fewer variables. He did not find macro‐level vulnerability assessments particularly enlightening because of the complexity of factors involved at higher scales (Hinkel, 2011). The VCI accordingly is also an instrument for vulnerability assessments at the local scale, which could paint a meso‐scale picture if extensive enough.

Wisner et al. (2004) formulated a pressure and release (PAR) model to illustrate the progression of vulnerability from structural root causes to dynamic pressures to unsafe conditions. The PAR model is in the venerable chain of explanation tradition in political ecology where everyday geographies of power imbricated with the political economy are implicated in local‐level environmental problems, including vulnerability to hazards (Van Dyke, 2015). The vulnerability picture conveyed by the VCI can be theoretically interpreted through the PAR lens, such as to capture the institutional and structural drivers of vulnerability.

Mustafa et al. (2011) formulated the VCI as a theoretically‐driven, but empirically‐informed assessment tool, which could be used to acquire household‐ and community‐level vulnerability profiles quantitatively. It identifies 12 drivers of vulnerability, which are divided into three categories, following Anderson and Woodrow (1989): material (education, exposure to hazard, individual assets, and livelihoods); institutional (earning members in a household and membership of a disadvantaged minority, employment and minority status, extra‐local kinship ties, infrastructure, social networks, and warning system); and attitudinal (empowerment and knowledge). The original formulation of the vulnerabilities and capacities matrix of Anderson and Woodrow (1989) is one of the most commonly used instruments for performing qualitative participatory vulnerability assessments across the Global South (see, for example, ActionAid, 2005), and hence the VCI builds on the strengths of this tested mechanism. There are four versions of the VCI: rural household‐ and community‐level and urban household‐ and community‐level. The rural household‐ and community‐level VCI matrix is used here (see Table 2). The rural community‐level VCI roughly addresses the same parameters as the household‐level VCI, with some modifications to the relative weights and the earning members in the household variable replaced by community unemployment rates (Mustafa et al., 2011).

**Table 2:** A composite VCI for the household level in rural areas

		Vulnerability	Capacity
	**Material vulnerabilities**	**35**	
1	Income source: start value	10	
	• Start value represents 100 per cent dependency on a local‐level productive asset, such as fisheries, land, and small shops.		
	• Add ‘2' to the score if the income sources are unstable, such as daily labour.	+2	
	• Subtract ‘2’ if local income sources are stable and insensitive to a local hazard.		−2
	• Lower the score by ‘1’ for every 10 per cent of non‐local income reported.		−1 per
2	Educational attainment: start value	5	
	• Start value represents no member of the household being literate.		
	• Lower the score by ‘1’ for every five years of schooling of the most educated male member of the household.		−1 per
	• Lower the score by ‘2’ for each female member's five years of schooling.		−2 per
3	Assets: start value	8	
	• Start value represents no immediately fungible assets, such as animals, farm implements, household items, jewellery, and savings.		
	• Lower the score by ‘1’ for every PKR 20,000 of fungible assets.		−1 per
	• Will have to be calibrated empirically.		
4	Exposure: start value	10	
	• Start value represents location in a high likelihood impact area relative to the prime hazard, such as household within the 10‐year floodplain.		
	• Lower the score by ‘1’ for every level of decreased impact likelihood between household location and high impact likelihood area, such as subtract ‘1’ for each 10‐year floodplain delineation.		−1 per
	• Lower the score by ‘1’ for each instance of hazard mitigation, such as the building of a house on higher plinth for floods.		−1 per
	**Institutional vulnerability**	**50**	
5	Social networks: start value	10	
	• Start value represents no household memberships of caste, ethnic, professional, or religious organisations.		
	• Lower the score by ‘1’ for each organisation to which a household member belongs.		−1 per
	• For each organisation that has provided assistance in the past, lower the score by twice the proportion of respondents reporting the organisation to be efficacious.		−2* (prop) per
6	Extra‐local kinship ties: start value	5	
	• Start value represents no extra local kinship ties.		
	• Lower the score by ‘2’ for every immediate family member living extra‐locally.		−2 per
	• Lower the score by ‘1’ for every non‐immediate family member living extra‐locally.		−1 per
7	Infrastructure: start value	16	
	• Start value represents a lack of access to electricity, healthcare, roads, tele communications, and water.		
	• Lower the score by ‘4’ if the household located has nearby access to a sealed, all‐weather road.		−4
	Or		
	• Lower the score by ‘2’ if the household located near a seasonal road.		−2
	• Lower the score by ‘2’ if the household has access to clean drinking water.		−2
	• Lower the score by ‘4’ if the household has mobile telephone coverage.		−4
	• Lower the score by ‘4’ if the household can access a local medical facility.		−4
	• Lower the score by ‘2’ if the household has access to electricity.		−2
8	Warning systems: start value	4	
	• Start value represents a lack of a warning system, or a warning system of which the household is not aware of or does not trust.		
	• Lower the score by ‘4’ if the warning system exists and is trusted.		−4
9	Earning members in a household: start value	5	
	• Start value represents a household composed of only one earning member.		
	• Add ‘5’ to the score if a single parent‐headed household.	+5	
	• Lower the score by ‘1’ for every additional earning member.		−1 per
10	Membership of disadvantaged lower caste, religious or ethnic minority	+5	
	**Attitudinal vulnerability**	**15**	
11	Sense of empowerment: start value	10	
	• Start value represents no participation in or access to leadership structure at any level.		
	• Lower the score by ‘10’ if the household is a self‐declared community leader and/or has declared active participation in community decision‐making.		−10
	• Lower the score by ‘10’ if the household has declared access to a regional or national leadership structure.		−10
12	Knowledge: start value	5	
	• Start value represents a lack of knowledge of potential hazards.		
	• Lower the score by ‘1’ for every type of hazard and related potential impacts accurately listed by respondents.		−1 per
	**Total vulnerability score**		–
	**Total capacity score**	–	
	**Combined vulnerability and capacity score**	–	
	**Highest possible vulnerability and capacity score**	**100**	

**Notes:** ‘per’ in the capacity column indicates ‘per’ source of income, in this case, or the unit specified; (prop) per indicates the number multiplied by the proportion of respondents. **Source:** Mustafa et al. (2011).

The 12 drivers of vulnerability, which are a part of the architecture of the VCI, are identified according to their significance in the vulnerability literature, or what Hinkel (2011) calls the deductive approach. The weights assigned to the variables are based on the normative judgements of the authors and their distillation of the vulnerability literature. While the universe of vulnerability drivers is practically infinite, the assumption of the VCI is that the 12 drivers that are part of it will explain a preponderance of the variance in household‐ and community‐level vulnerability. There is a proliferation of vulnerability indices (see, for example, Luers et al., 2003; Vincent, 2004; Rygel, O'Sullivan, and Yarnal, 2006; Bosher, Penning‐Rowsell, and Tapsell, 2007; Khan and Salman, 2012), but this is one of the few instruments that has been peer reviewed. Accordingly, this instrument was selected for empirical testing in the vulnerability assessment exercise.

It is important to note that the VCI is a tool for *comparative* analysis rather than an absolute indicator of vulnerability. A higher VCI score would mean a higher level of vulnerability and vice versa. In interpreting the results of the VCI survey, though, one must bear in mind five points: (i) the index is taking a static snapshot of what is essentially a dynamic process; (ii) it is based on the experience of South Asia and may have to be modified for different contexts; (iii) the VCI score is for comparative purposes only and does not mean anything by itself—it is important, therefore, that weights are applied consistently across field sites; (iv) its simplicity is its strength but also its weakness in that it will inevitably miss some complex interlinkages; and (v) it must not be used alone but in conjunction with qualitative information.

**Figure 2:**
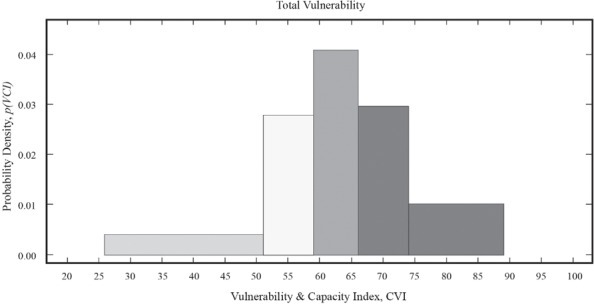
Distribution of categories of vulnerability: resilient, low, moderate, high, very high, and extreme **Source:** authors.

The above limitations notwithstanding, VCI scores can produce a simplified snapshot of differential vulnerability that can be an invaluable tool for action. Even the limitation of interpreting the VCI scores by themselves can be overcome *partially* with a big enough sample (n=1,259 in this case, for instance) and by running the Jenks (1967) natural breaks optimisation method to classify the data into groups of high, medium, low, and resilient populations. The Jenks method classifies the data by maximising the variance between categories and minimising the variance within categories. Figure 2 shows the results of the Jenks routine as applied to the VCI data.

The VCI score boundaries for the categories as outlined in Figure 2 are as follows:

- resilient, 0–37;
- low vulnerability, 38–50;
- moderate vulnerability, 51–59;
- high vulnerability, 60–66;
- very high vulnerability, 67–74; and
- extreme vulnerability, 75 and above.

This empirically‐derived global classification can serve as a further simplified tool with which policymakers can interpret the results of any VCI exercise. The remainder of the VCI scores presented in the next section should be interpreted with these categories in mind.

## Results

### Vulnerability across agro‐ecological/livelihood segments of Sindh Province

**Figure 3:**
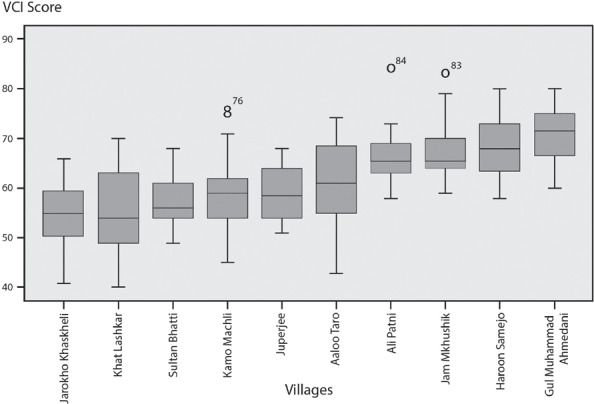
Box plots for the HH‐VCI scores of villages with canal irrigation and fresh groundwater VCI Score **Source:** authors.

**Figure 4:**
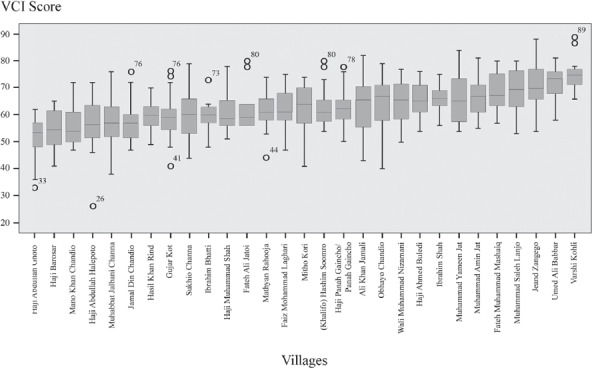
Box plots for the HH‐VCI scores of villages with canal irrigation and saline groundwater VCI Score **Source:** authors.

**Figure 5:**
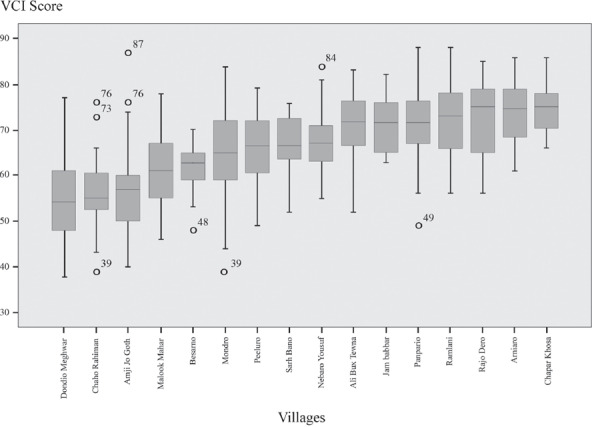
Box plot for the HH‐VCI scores for agro‐pastoralist villages VCI Score **Source:** authors.

**Figure 6:**
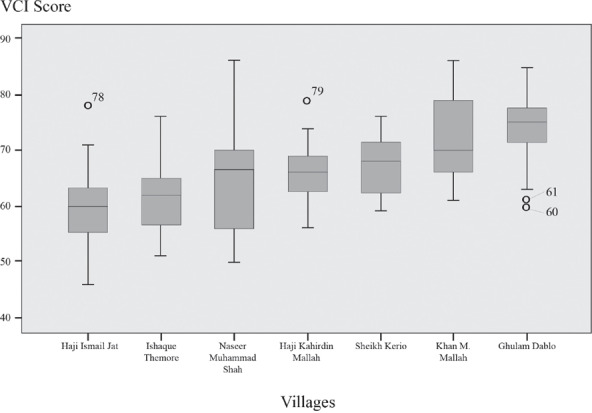
Box plot for the HH‐VCI scores of fishing villages VCI Score **Source:** authors.

The VCI yielded considerable variance between household‐ and community‐level level data points. The average household‐level vulnerability scores for communities were significantly correlated with the community‐level VCI (Comm.‐VCI) scores calculated using the rural community‐level VCI template. The Pearson's correlation test between the two numbers indicated a significance of 0.000 and a Pearson's correlation coefficient of 0.603, denoting a significant but moderately strong correlation. The moderate strength of the correlation is a function of the internal diversity of household‐level VCI scores within communities (see Figures 3, 4, 5, and 6). The point, however, is that if one is pressed for time and resources and hence is not able to perform a household‐level VCI (HH‐VCI) analysis, quick use of the Comm‐VCI can generate good enough results (see Mustafa et al., 2011). The types of variance reflected in Figures 3, 4, 5–6 could help decision‐makers to prioritise communities for DRR and vulnerability reduction interventions.

#### Drivers of vulnerability: access to irrigation water and low social capital

It is interesting to review briefly some representative households and what is known about them to illustrate why they fall where they do on the HH‐VCI spectrum. Many of the single mothers, widows, and divorcees in the sample tended to have very high VCI scores, partly because of the patriarchy‐driven social stigma attached to WHHs, and partly because of the fragile livelihoods of such households. Being alone, however, was not enough for a high vulnerability score—for instance, one of the highest VCI scores, 86 (extremely vulnerable), was for Bhago, an 80‐year‐old illiterate widow from the agro‐pastoralist village of Mondro in Tharparkar District, who has a house full of children and grandchildren. Her 46‐year‐old son is in prison, while her 45‐year‐old daughter‐in‐law is a daily wage worker and a livestock trader. Her grandson, aged 14, manages livestock for local landlords and her granddaughter, aged 12, does some embroidery work for money. Nobody in the household has had any schooling; they do get some supplementary income from the government's social protection programme, the Benazir Income Support Programme. This household has high vulnerability because of no reported extra‐local kinship ties, which could be a source of help, no awareness of any warning system, and no access to any infrastructure. It is also highly indebted because of food expenses, something that the VCI does not address.

Almost all WHHs are extremely vulnerable (modal VCI=79, n=157), yet there are instances of MHHs that are also extremely vulnerable (modal VCI=63, n=1,202), such as Mahmud from the coastal island fishing village of Ghulam Dhablo (Comm.‐VCI=70) in Thatta District. Mahmud is a young man of 25 with a 23‐year‐old wife and three children. While his wife is a full‐time homemaker, Mahmud is a fisherman, who used to operate locally, but now has to travel up to 90 kilometres from the coast to catch any fish, owing largely to overfishing by commercial trawlers in the coastal zones and the contract fishing allowed in coastal waters since the late 1990s (Gowdy and Salman, 2011). He owns his fishing boat and a net, which are his only assets. What distinguishes him in particular is that he is estranged from his community and has no access to any associational life (that is, membership of any formal or informal groupings) or leadership structures. His vulnerability is primarily a function of the very high vulnerability of his community (modal VCI=73). Many of the factors contributing to Mahmud's vulnerability are common in his village, with the exception of his household's especially low social capital.

At the lower end of the spectrum, Abdul Rahim from Khat Lashkar in Dadu District has a VCI score of 40. Khat Lashkar is a canal irrigated village at the head of a canal with fresh groundwater. Rahim lives with his three brothers, wife, and mother. His wife has a university education and his mother also is educated. He has a government job as a stenographer in the country's capital city, Karachi, while one of his brothers has an agricultural income, and the other two are unemployed. The household is well equipped with electrical appliances, such as a flat iron, refrigerator, and television, and owns a motorbike for transportation. The household did not report any associational membership, or extra local kinship ties, but it did report good linkages with community and local leadership structures. Comparable case studies were found, mostly among households in canal irrigated areas with a rich associational life, multiple sources of income, and access to relatively good infrastructure, as well as low vulnerability scores. In agro‐pastoral villages too, case studies were found of households with low vulnerability, largely on account of diversity of income and high social capital. Again, the canal irrigated geography is a historic artefact,^2^ determining lower vulnerability for some, and mere survival and high risks for others. The British colonial authorities deliberately settled those who they perceived to be loyal to the Empire on large estates at the head of water courses. These farmers went on to become the leaders of the rural society, owing their privilege to the British. The inability to cope with the present (and high vulnerability scores) is not just a matter for those excluded altogether from the Canal Colonies, but also is an issue for those who are within them but are landless or at the tail end of the canals.

Although the aforementioned sample household studies can validate the types of VCI scores derived from the survey, one must be cautious in not over relying on these scores for an assessment of gendered vulnerability. The VCI does a competent job of capturing gendered vulnerability at the higher end of the spectrum, but, at the lower end, the scores often can obfuscate higher gendered vulnerability within households. This point will be thrown into sharper relief as the VCI scores at the community level are validated using qualitative information.

#### The tale of communities beyond households and their VCI scores

This subsection examines four villages, representing each of the agro‐ecological/livelihood zones. The profiles illustrate why there is a difference in the Comm.‐VCI between the communities.

In the inland fishing village of Haji Khair Din Mallah on Lake Manchar in Dadu District (Comm.‐VCI=68, HH (household)‐mean=67), the majority of people previously lived on houseboats. In fact, there were between roughly 45,000 and 50,000 people living on the lake until 1990. By 2010, the population had dwindled to less than 20,000, mostly because of the World Bank‐funded construction of the Right Bank Outfall Drain, to channel agricultural effluents and municipal waste from low‐lying lands and communities on the right bank of the River Indus to the Arabian Sea. The project as a whole is ongoing, but the Main Nara Valley Drain has been dumping waste into Lake Manchar since 1996 (Birwani and Noshirwani, 2014; Ghaus et al., 2015), destroying fisheries and diminishing the quality of the water, with disastrous ramifications for the community:

*We sold out our boats in time of need, when we could not get a livelihood from them. Now if anyone wanted to buy a boat it would be nearly impossible because it costs two to three lacs [hundred thousand] rupees. Sometimes we struggle for one meal a day when we get no fish from water. As we are Mallah [boat people/sailors], we only know the fishing profession, and have no experience or skills for any alternate profession* (FR (female respondent), Haji Khair Din Mallah).

*Previously we did fishing with males in every activity at equal level and had more economic activities and had very prosperous life* (FR, Haji Khair Din Mallah).

**Figure 7:**
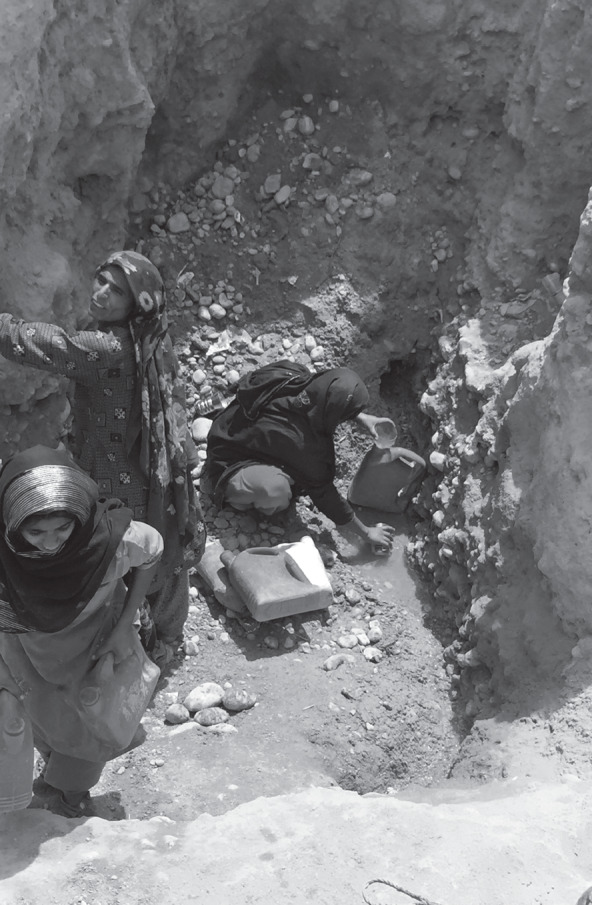
Women scooping up brackish water from a groundwater source **Source:** authors.

As the quotations illustrate, not only was an entire way of life lost as a consequence of the changes brought about by the Main Nara Valley Drain, but also they had deleterious effects on gender relations in communities, not least of which was the burden of fetching low‐quality water (see Figure 7). Whereas women were engaged in livelihood activities in the past, now they are restricted to the domestic sphere. Hence, the high vulnerability score of the village is not separate from the question of development, or from the gendered impact that such transformations have on livelihoods and mobility.

Things are comparably bad in Ramlani in Tharparkar District, a moderate‐sized agro‐pastoral village in the middle of the desert with no access to a motorable road, electricity, or health facilities (Comm.‐VCI=79, HH‐mean=74, HH‐mode=75). The nearest health facility is some 60 kilometres away, and the primary school in the village is not functional because there is no teacher. In the desert environment of Ramlani, the lives of women and children largely centre on collecting water. A government‐installed tube well in the village yields only brackish water, and the nearest fresh water source is about eight kilometres away. Women and children are largely responsible for obtaining water, forcing them to walk to the source once a day in the winter, and up to three times in the summer. There is a significant amount of seasonal outmigration of men to irrigated areas in other parts of Sindh Province or to cities such as Hyderabad and Karachi. In their absence, the women reported much higher workloads, although they also enjoyed greater decision‐making autonomy. Sometimes women also have to migrate with their able‐bodied men in search of work and water, rendering the elderly and the sick in the community particularly vulnerable.

Not all agro‐pastoral villages fare as badly as Ramlani. The people in the village of Besarno (Comm.‐VCI=57, HH‐mean=62, HH‐mode=63) have managed to mitigate their vulnerability by diversifying their income through employment with the government and the military. As a result, they report having access to local leadership structures, as well as to three deep wells for fresh water. A boys’ and a girls' primary school are also present in Besarno.

In canal irrigated villages with fresh groundwater, things are significantly better, especially if they are at the head of a canal. In Khat Lashkar (Comm.‐VCI=46, HH‐mean=55, HH‐mode=49), the more than 2,500 residents mostly live in brick and cement housing and have access to amenities, including electricity, in‐house hand pumps, propane gas, schools, and a motorable road, although there is no healthcare facility. The nearest basic healthcare unit is between two and three kilometres from the village. Most of the respondents from Khat Lashkar were university educated, engaged in the teaching profession, and had employment with the government and in the private sector, in addition to a customary agricultural source of income. Moreover, this village had relatively higher levels of educational attainment than all of the other villages in the sample—53 per cent of females in the sample were educated).

The increased well‐being and financial capital of the household translates into more control over women's mobility, with that control serving as ‘symbolic capital’ aimed at displaying the household's increased status (Siegmann and Thieme, 2010). As one respondent pointed out:

*Most neighbourhood females were working in the field for agriculture, but since the past 15 to 20 years the females in our tribe have changed. Our livelihood has changed and therefore we are not working as agricultural labour . . . Now we are more religious and our males do not allow us to move about and insist that we cover with veil* (FR, Khat Lashkar).

Many of the women attending the FGD in this village were wearing a burqa, which is not a part of traditional dress in Sindh. Greater prosperity and education, ostensibly contributing to lower vulnerability, have gendered outcomes: women face less drudgery, but the control over their bodies (decreased mobility, increased gender segregation, and the process of seclusion known as *purdha*) is a symbol used to portray the enhanced economic status of the household. Such outcomes may contribute to greater gender differentiated vulnerability, which the raw VCI score is unable to capture in this instance.

In irrigated villages with saline groundwater at the tail end of canals, things are much different. The highly vulnerable Varshi Kohli (Comm.‐VCI=73, HH‐mean=74, HH‐mode=75) is in Badin District and all of its approximately 450 residents are Hindu migrants from neighbouring Nagarparkar. Apart from a boys' primary school and a seasonal motorable road, the village has no infrastructure or facilities, such as electricity. Some hand pumps are functional, but they mostly deliver brackish water. The residents are largely associated with wage labour in the area, although some people still maintain landholdings in Nagarparkar, from which they derive an income. The village is quite close to the Left Bank Outfall Drain (LBOD),^3^ which is a source of flooding. Flooding in 1984, 1994, and 2011 displaced the village population, which was forced to return temporarily to Nagarparkar. Ironically, while they migrated because of a lack of water, they are periodically displaced because of too much water. Other canal irrigated villages with saline groundwater, however, have fared better. Mano Khan Chandio (Comm.‐VCI=49, HH‐mean=54, HH‐mode=51), located at the head of a canal, has high levels of educational attainment among males, and diversified livelihoods. Yet it too displays forms of gendered vulnerability: only 11 per cent of women are educated and suffer from restricted mobility as an outcome of increased prosperity.

**Figure 8:**
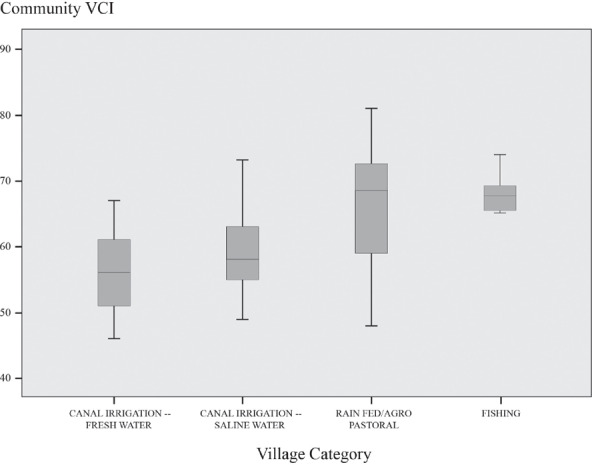
Difference in aggregated household VCI scores between different agro‐ecological/livelihood zones Community VCI **Source:** authors.

The variance in the Comm.‐VCI among the illustrative communities discussed above is principally on account of diversity of income and access to levers of power, as well as, to a lesser extent, access to different types of infrastructure. While exposure to different types of environmental hazards is relatively similar across the villages, how each of them is positioned within the political economy of Pakistan seems to be critical to their vulnerability profile. The canal irrigated villages, regardless of the quality of groundwater, appear to have significantly lower VCI scores—which seem to increase the further down the canal is the location of the village—than the agro‐pastoralist or fishing villages (see Figure 8). Nevertheless, even here, one should still be cautious about jumping to technologically‐driven conclusions about the utility of canal irrigation in mitigating vulnerability (Mustafa, 2002; Taylor, 2015). Location along the canals is the product of a very historically contingent process, which often was an outcome of deliberate social engineering undertaken by colonial and post‐colonial state authorities (Gilmartin, 1994; Mustafa, Akhter, and Nasrallah, 2013). In addition, the lack of significant difference in the community VCI values between saline and fresh groundwater is a surprise, given that the canal is the sole source of water for agriculture and domestic purposes, and the villages do not have the option to supplement canal water with groundwater—we expected vulnerability to be much higher than it is because of this.

These case studies convey how the Comm.‐VCI score can be indicative of the relative vulnerabilities of the communities, yet they also underline the need to go beyond the numbers to understand the specific drivers and configurations of community vulnerability. The numbers point to something interesting happening, but it is the qualitative information that provides the explanation for the vulnerability.

## Climate vulnerability: from numbers to narratives

Despite the thrust of the argument so far being on socially‐determined vulnerability, the biophysical effects of climate change are not irrelevant to the changes occurring in Sindh Province. As the biophysical effects are mediated by societal forces that amplify or diminish the impacts, it is difficult to pronounce judgement on whether or not a particular ramification is a direct consequence of climate change. As one respondent pointed out:

*June 15 was the time when the rains would always come, now it is unpredictable. There is more heat and increase in temperature. June and July were the summer months but now there are longer periods of heat. In the past in April and May there was no heat* (MR (male respondent), Mondro, Tharparkar).

The two most important instances of climatic change reported by the communities were temperature extremes and shifts in the timing of the rains, impacting agricultural and pastoral rhythms. These changes seemed to be coupled with people's perception of alterations to flora and fauna. The effects of these changes, though, manifested in a rural economy itself in a state of flux. People have switched their agricultural production from onions, for instance, to cotton, increasing demand for water, and their housing architecture from adobe mud houses (which are naturally insulated and hence cool in summer and warm in winter) to electricity dependent burnt‐brick and concrete houses. Furthermore, environmental hazards such as floods were considered to be the most damaging climate‐related hazard. In a highly manipulated basin like that of the Indus, floods are mostly a function of human transformation (see, for example, Mustafa and Wrathall, 2011).

Regardless of the biophysical versus social sources of climatic stress, the effects are definitely gendered, starting with the lack of early warning systems. As two respondents observed:

*We [women] never get any prior information [such as about cyclones] from any source. Now males are alert and they are getting news from the radio* (FR, Gul Muhammed Ahmedani, Badin).

*If we face anything like a disaster it could be a difficult situation for women to shift to another place as being part of rigid and customary tribe we will not be allowed to shift to camps, since we are not allowed to face any alien or outsider* (FR, Juber Jee, Dadu).

Once an extreme event is happening, in a society with gendered lines of power and livelihoods, the signs of it are also inevitably gendered (see, for example, Sultana, 2014; Mustafa et al., 2015). Gendered power relations frequently are internalised, and therefore are much harder to confront. As one respondent explained:

*Domestic violence in this tribe is common and women take it as their life routine and they have no objection to it. It is man's honour and it is his right to beat women since women are not very intelligent, so they make mistakes and men always correct it, so sometimes they beat them* (FR, Juber Jee, Dadu).

Vulnerability is also embedded in the fragility and stresses of ‘normal’ life (Hewitt, 1983; Wisner et al., 2004). The differentiation in workloads by gender is one example of that fragility in the field study villages. In more well‐to‐do villages, there are increasing limits to female mobility, whereas women in poorer communities bear greater workloads and longer working hours. As two respondents noted:

*Pattern of activities is we wake up at 5.00 a.m., start day with prayer and then cooking, washing, skilled work (knitting fish net, crochet work, tailoring). We have some livestock so collect fuel wood, carry out collection and drying of cow dung. After dinner then bedding and if [. . .] electricity allows us so we also chat and do some skilled work late at night* (FR, Manchar Lake, Dadu).

*We fetch water from the well. We go twice to fetch water for household chores, in the morning and evening, and each time it takes 2–2.5 hours. If anyone has livestock they have to go more often* (FR, Besarno, Tharparkar).

These working hours are also inflected by the increasing monetisation of the previously moral economy, in which the moral imperatives of mutual obligation were the basis of social interaction (Appadurai, 1984). As one respondent observed:

*In this area there is only one hand pump in another village, but if anyone takes water from that, the people of that household have to pay PKR 500 per month for the water. Since we cannot afford PKR 500 we have to walk for two hours to get water* (FR, Ibrahim Bhatti, Thatta).

Three key third‐order impacts are worth mentioning in the context of the gendered impacts of changes in the rural society of Sindh Province, pertaining to health, labour migration, and nutrition. Many communities described ensuring adequate nutrition as difficult even in ‘normal’ circumstances, as income from agriculture and livestock has fallen, the natural flora and fauna have been depleted, and food inflation has increased. While the decline in food quality and quantity affects whole families, it is women who appear to bear the heaviest load, and who are most vocal about this concern:

*In the past there was less heat and we spent less. Now things are very expensive. We only eat vegetables. We cannot afford meat or eggs, it is too expensive, but sometimes the men eat eggs* (FR, Dondio Meghawar, Tharparkar).

*The food we eat has changed; we had vegetables, now we eat red chillies and roti (bread). Because of the drought we cannot grow food* (MR, Amji Jo Goth, Tharparkar).

*I had more than 35 goats and a couple of cows, but all those drowned in the recent floods. Now it is impossible for me to even buy one goat* (FR, Haji Abdullah Halepoto, Badin).

*It is very tough to meet needs on a daily basis, with the limited amount for food . . . especially in this time of high inflation like a kilogram of flour costs PKR 40–45. Due to shortage of food, first we serve to children and males; few times we females have to survive without meals* (FR, Jam Babbar, Tharparkar).

The health of the study communities paralleled nutrition: people appeared to be suffering already from health‐related issues such diarrhoea, fever, gynaecological issues, jaundice, kidney stones, liver problems, malaria, malnutrition, and skin rashes. The situation was exacerbated by the increase in women's workload, limitations on their mobility, and changes in the healthcare system from a more traditional low cost setup to a more commercial Western medical arrangement. In times of natural disaster, circumstances deteriorate further, with particularly dire consequences for women:

*For maternity problems there is no lady health worker or trained dai [midwife] available, so in any emergency we rush to Piyaro Khan town where we get [a] female doctor; it is 10 kilometres away and in general we get treatment which costs around PKR 1,000, but in delivery cases and other maternity cases they charge PKR 5,000 to PKR 7,000, which is not affordable by any poor person* (FR, Juber Jee, Dadu).

*Some women get gynaecological problems, which are chronic as they could not afford to go to hospital frequently* (FR, Gul Muhammed Ahmedani, Badin).

*We cannot move without permission of our males; in emergencies too we have to take a male otherwise we are not allowed to take them [sick women] for health emergency* (FR, Khat Lashker, Dadu).

As the quotations illustrate, one of the more insidious consequences of male outmigration, in addition to the increase in women's workload, is not being able to access healthcare. To approach anyone other than a husband for transport to health facilities for gynaecological problems, for instance, would be unthinkable, meaning the women just suffer in silence.

The ethnographies presented above highlight specific aspects of gender vulnerability differentially affecting people's capability to cope with climatic and socioeconomic changes. A lack of awareness of an early warning system, healthcare, nutrition, monetisation of the economy (and hence inflation), and women's workloads and limits on their mobility emerge as the main contributors to gendered vulnerability. Almost all of these factors are embedded in the cultural ethos and political economy of Pakistan, which accentuates experiences of heat stress and other hazards, such as floods. The narratives of vulnerability complement and nuance the findings of the VCI, for instance, the VCI can capture whether or not there is an early warning system and if a respondent is aware of it, but not differences in knowledge of it between men and women. The VCI may capture the proximity of healthcare services, but not their quality or affordability. This is true of many other factors that are not part of the architecture of the VCI. Extraneous variables outside of the VCI architecture end up affecting those that are a part of it. For example, poor health may impact livelihoods, which the VCI does capture, but the chain of causation ultimately must be disentangled using qualitative information.

There is evidence regarding the changing biophysical aspects of people's lives in the study region, but it is unclear how independent are the impacts of the changes, and on balance how much more important they are than the social context of patriarchy, the transition to a monetised economy, and the commercialisation of agriculture and fishing. The next section contains some reflections on the efficacy of the methodology that helps to uncover these stories and their substantive valence.

## Conclusion: climate challenges are yesterday's news

The variance in comparative vulnerability across the household and community scale is reflected well in the VCI scores. What is more, these are confirmed by the customary (in the critical realist and political ecological tradition) qualitative vulnerability assessment. Study field staff (whose level of education ranged from high school to some level of university) were able to understand and calculate the VCI scores for households and communities, while at the same time documenting the basis for them, as well as household and community profiles. This provides confidence that the instrument is not only robust in conveying the variance in vulnerability, but also that it is easy to use in most field environments, especially in the Global South. From DRR to climate change adaptation to even disaster relief, mapping VCI profiles would help in prioritising and targeting the most vulnerable households and communities. In terms of post‐disaster relief provision in particular, the lack of pre‐existing knowledge of the vulnerability profiles of communities causes chaotic delivery, as some communities end up getting too much, and others receive nothing (see, for example, Ozerdem, 2006). Long‐term developmental interventions similarly could use the tool to comprehend the key drivers of vulnerability and inform interventions on how to address them.

Narrative vulnerability is important and has enriched understanding of the concept and its drivers. However, there is a pragmatic imperative to move from narratives to numbers. VCI is a useful tool with which to satisfy that imperative. Having moved to numbers, though, one inevitably must return to narratives to understand the stories behind them. Numbers are not a substitute for, but rather a supplement to, narrative forms of vulnerability assessments. This is especially true when it comes to gendered vulnerability. The VCI instrument is good at highlighting vulnerability at the higher ends of the spectrum, but the hidden gendered vulnerability in the middle reaches of the VCI spectrum is only discernible when the numbers are combined with the narrative vulnerability assessment.

This paper has underlined, too, that the very urgent present of the survey respondents and communities is defined by extremes of deprivation and marginalisation, an inability to cope, and differential vulnerability. It has emphasised that the lack of coping capacity and vulnerability are not a function of exposure to climatic extremes, but instead of how households and communities are positioned within the political economy of Pakistan. More favoured access to irrigation water at the head of a canal in fresh groundwater zone is a distinctly human artefact, as is the degradation of Lake Manchar owing to overfishing and a World Bank‐funded project, or the hazards emerging from the LBOD. Vulnerability is more a function of historico‐political economic factors than any biophysical changes wrought by climate change, and is likely to remain so. Women's enhanced vulnerability is a function of the patriarchal ethos of rural Sindhi society, as well as of increased workloads borne of male outmigration, monetisation of the economy, and poverty. At times, perversely, the drudgery for more affluent households is replaced by greater restrictions on women's mobility as a source of symbolic capital. The VCI helps to spotlight the urgent present of differential vulnerability, while the ethnographies reveal the gendered aspects of it, especially in low‐ to mid‐range vulnerable households, where the apparent picture of less vulnerability hides higher gender‐based vulnerability.

This paper has proposed and validated a methodology that may help to communicate that urgent present, as viewed using a vulnerability lens, to the policy realm. More than 30 years of vulnerability research has helped to ensure the term's inclusion in the policy lexicon, but actual policy informed by rigorous vulnerability analyses remains elusive. Hopefully this methodological engagement with vulnerability to climate change helps to address this practical and theoretical gap.

## Acknowledgements

The fieldwork for this paper was funded by an International Development Research Centre grant (number 106857‐002). The support and guidance of Dr Khalida Ghaus, the Executive Director of the Social Policy and Development Centre, is gratefully acknowledged. Asif Iqbal, Tabinda Areeb, and Naveed Amir were instrumental in the conduct of the research and in providing useful feedback.

Dr Gioli's contribution to this work was made thanks to the support of the Himalayan Adaptation, Water and Resilience consortium under the Collaborative Adaptation Research Initiative in Africa and Asia and the financial support of the Government of the United Kingdom's Department for International Development (DFID) and the International Development Research Centre in Canada. His research was also partially supported by the core funds of International Centre for Integrated Mountain Development (ICIMOD) contributed by the governments of Afghanistan, Australia, Austria, Bangladesh, Bhutan, China, India, Myanmar, Nepal, Norway, Pakistan, Switzerland, and the UK.

The views expressed in this paper are those of the authors and do not necessarily represent those of DFID or the International Development Research Centre or its Board of Governors. In addition, they are not necessarily attributable to ICIMOD and do not imply the expression of any opinion by ICIMOD concerning the legal status of any country, territory, city or area of its authority, or the delimitation of its frontiers or boundaries, or the endorsement of any product.

## Endnotes

1 To understand the historical reasons for contemporary vulnerable geographies in the Canal Colonies of Pakistan, see, for example, Michel (1967), Ali (1988), and Gilmartin (1994, 2013).

2 The LBOD is the result of a World Bank‐funded project to build a spinal main channel to drain brackish water from southern districts of Sindh Province on the left bank of the Indus River and to put it in the Arabian Sea.
